# Using Co-Design to Explore New Trends in Future Kitchen Designs: An Exploratory Workshop Study of College Students in China

**DOI:** 10.3390/ijerph20021550

**Published:** 2023-01-14

**Authors:** Zhidiankui Xu, Yu Wu, Yixi Bao, Jiawei Li, Zhengzhong Zhou

**Affiliations:** 1 School of Design, Jiangnan University, Wuxi 214122, China; 2 Academy of Arts and Design, Tsinghua University, Beijing100089, China

**Keywords:** Chinese college students, co-design, future kitchen design, health, well-being, user expectations

## Abstract

The current COVID-19 pandemic is exacerbating the challenges facing human society. The public is increasingly concerned about the health and well-being of individuals, families, and communities. To enhance human health and well-being, user expectations for the future need to be understood. The kitchen, a central area of a home, is closely related to healthy living. In this study, a series of seven exploratory workshops were held at a Chinese university using co-design to understand the expectations and thinking of Chinese college students about the future of kitchen design in terms of health and well-being. A methodological innovation was introduced in co-design workshops, where participants were asked to imagine, discuss, and sketch concepts together to stimulate creative design. A six-dimensional tentative model of future kitchen expectations, including 34 sub-themes, was constructed based on the data analysis to explore the expected characteristics of kitchens. These dimensions include intelligent technologies and interaction experiences, health and well-being, inclusivity and extensibility, ecosystem circulation and sustainability, emotional and meaningful experience, and spatial planning and aesthetic experience. The resulting model provides valuable insights into the expectations of future users, providing direction and systematic strategies for future kitchens along the six-dimensional characteristics. Future kitchens, if the younger generation is to adopt them, need to positively affect users’ lives and meet their health and well-being standards.

## 1. Introduction

The threat of the COVID-19 pandemic is increasingly exacerbating challenges for the entire human race and pushing us into a more distant future [1]. It affects various areas of human social well-being, such as international public health, business, economics, and education [2], leading to a slowdown in social development and significantly impacting human society. The impact of COVID-19 also poses a severe threat to people’s physical and mental health and well-being [3], e.g., by limiting people’s group activities and social behavior to reduce the spread of the disease. It causes various psychological problems, such as anxiety, confusion, loneliness, depression, boredom, and anger [4]. Thus, there is a growing concern about the health and well-being of individuals, families, and communities. Understanding and focusing on users’ actual needs and desired futures is essential to enhancing human health and well-being, which helps foster innovation in user experience research and guides the fuzzy front-end stages of the design process. Currently, people often need to work at home in China because of the epidemic. More extended stays at home have led to an increasing focus on the interior space at home and a higher demand for quality of life.

The epidemic is a painful reminder that design decisions need to be informed by future possibilities in the face of complex sustainability challenges, and that design is responsible for the unknown future. It requires all designers to face the fact that the future is unknowable and to consider the various possibilities [5]. Designers are always imagining the future and what it might hold [6], exploring future trends, and seeking new ways to improve the quality of people’s health and well-being [7]. However, there is uncertainty about exploring the future, and what is designed in the present may be used in the future under conditions beyond the designer’s control [8]. Reeves et al. (2016) proposed that every current action is a movement into the future, which helps shape it but does not determine it. However, even the most mundane acts will fail if the desired results are not achieved [5]. One of the reasons for this lack of progress is a lack of imagination, and determining multiple future possibilities and how they can be achieved is a critical process in bridging this imagination gap [9]. Therefore, in exploring the future, designers need to comprehensively understand their target users and their needs, and it is vital to maintain empathy [10]. At the same time, it is essential to focus on users’ actual demand for future designs and to stimulate their imagination, allowing them to fully express their creativity and imagination. However, most current research on user experience has focused on their past and present needs, while research on users’ future needs is still scarce [6]. Related research shows that users seem to have difficulty expressing their expectations of new products [11]. To address this challenge and stimulate the imagination of users to express their expectations, further research is needed.

Recently, young people have attracted more attention and have been recognized as the driving force behind new cultural trends and attitudinal shifts [9]. The positive impact of involving young people in innovation has been demonstrated [12], and involving them in the innovation process and adopting their perspectives can have a positive impact on addressing current issues regarding the environment, health, well-being, and injustice in society [13,14,15]. Young people can increase their self-efficacy and ability to take charge and generate new ideas by participating in co-creation [16]. College students, as a special and representative group of young people, are the mainstay of the future middle class; they are receptive to new ideas and have higher expectations for the quality of life in the future. They are highly educated thinkers and communicators, are creative and imaginative, and have great potential for development [17]. College students are highly concerned about health and environmental issues, are better equipped to promote mental health, and can achieve higher levels of health literacy [18,19].

### 1.1. The Design, Health, and Well-Being of the Kitchen

Recently, kitchen design has become a research hotspot. The kitchen has always played a central role in our homes [20,21], with many interactions occurring on a daily basis. It is a place where new technologies and traditions meet, as well as a place where family health and well-being are nurtured. It is closely linked to people’s healthy diets and lifestyles. The kitchen serves a social purpose and is multifunctional [22]. It needs to be comfortable and functional and provide a better user experience, allowing people to express their emotions and share their lives.

Current research in kitchen design focuses on the user’s mental health and well-being, human physical health and ergonomics, and the optimization and enhancement of the user experience arising from technological advances.

Some studies on mental health and well-being in the kitchen have focused on communication and well-being at home, using positive psychology as a guiding framework for community-based family interventions, applying it to positive communication in family cooking and dining, and conducting the development, implementation, and evaluation of the Happy Family Kitchen I (HFK I) and Happy Family Kitchen II (HFK II) [23,24]. Sun and Wu (2015) conducted research on the smart kitchen and applied motion analysis to a Tangible User Interface (TUI) design to enhance the experience of cooking together between couples and empower the cooking process with intimacy, communication, education, entertainment, and creativity [25].

Some researchers have argued that human physical health and ergonomics are crucial in kitchen design. Nour et al. (2018) conducted research on the problem of young people’s vegetable preparation and eating disorders in the kitchen [26]. Maguire et al. (2014) explored the personal health and ergonomic problems of older people in the kitchen and proposed more flexible and adaptable design strategies [22]. Wang et al. (2022) studied the relationship between the efficiency of the elderly’s daily activities and the spatial layout of the home kitchen and evaluated the moderating effect of cognitive function, helping to provide a basis for the future design and optimization of residential spaces [27]. Ficocelli and Nejat (2012) designed a cognitively assisted, interactive kitchen system for people with cognitive deficits that can help users overcome problems of attention and memory deficits [28].

With the progress of technology, the way we interact with the kitchen environment is constantly changing. Contemporary kitchens are equipped with a variety of intelligent kitchen appliances, which are controlled by new interaction modes such as voice, gaze, and gesture control [29,30]. Using these new technologies and interaction modalities has made the kitchen more convenient, accessible, and usable, helping users complete complicated tasks. Besevli et al. (2022) focused on gesture interaction and designed an information display named “2 Hand Touch” controlled by Hand-Specific on-Skin gestures (HSoS). This conceptual design is beneficial in terms of hygiene, time management, imminent control, and uninterrupted cooking practice [31]. Schuhmacher et al. (2018) designed a touch- and voice-controlled Visual Atmosphere Application (VAA), which uses visual projection and sound effects to change the kitchen’s atmosphere [32]. Sharath et al. (2018) developed an automatic cooking device for a kitchen that is based on AI technology and neural network training algorithms. It can automatically cook dishes according to users’ tastes and help users predict and manage their plans based on their cooking style [33].

This literature review shows that there are many unresolved problems in the current research on kitchen design, relating to, e.g., environmental protection and sustainability, spatial layout, and aesthetic experience, as well as new developmental trends arising from technological advances.

### 1.2. The Effect and Application of Co-Design

The co-design method is emerging as one of the ways to address global change and societal challenges [34]. The co-design is a “designerly” and “creative problem-solving” approach that seeks to explore “co-development problems and solutions” through joint inquiry and imagination [35,36]. Recent research has shown that co-design has been widely used and developed in many areas [37], and has had a positive impact. At the macro policy level, the public’s actual needs can be fully understood by co-designing policies and processes, such as in energy, the environment, and planning projects [38,39,40]. In knowledge-related research, academics use co-design in the context of knowledge production to facilitate the exchange and sharing of knowledge and enhance the value of research [41,42,43]. In product development, Hazard et al. (2016) has shown that user-friendliness and product acceptability can be promoted by co-designing and developing products with users, such as in developing household products [44]. Noorbergen et al. (2021) used the Agile methodology to co-design a smartphone app with dementia care partners and showed that the co-design process improved the acceptability of the product [45]. In the service field, researchers have explored co-design related to social and human health, aiming to improve trust between service providers and users and enhance service quality, efficiency, and compliance [46,47]. In the field of health care, Ward et al. (2018) discussed the value of co-design in healthcare, using a collective leadership intervention designed by healthcare professionals, patient representatives, and health system researchers to improve the performance of healthcare teams and the culture of patient safety [48]. Co-design has also been applied in areas such as urban development planning and public space design to increase the fit with human needs and behavioral patterns [49]. For example, Wu et al. (2022) used co-creative design approaches in the community design process to promote intergenerational integration [50].

Currently, there are few studies that use the co-design method to explore kitchen design trends. Therefore, this study uses the co-design method [51] to explore Chinese college students’ expectations of future kitchen design trends in the context of the COVID-19 pandemic. The advantage of using co-design is that it allows college students to participate in the design process in a more interesting way, contribute their creativity and ideas, enhance the sense of participation, stimulate group creativity [52], and provide new value to kitchen design research.

This study focuses on the issue of how kitchen design and people’s needs will change in the future after the COVID-19 pandemic. Based on the co-design method, this study innovates and expands by holding a series of seven exploratory co-design workshops. Each co-design workshop involved a four-step process in which participants were asked to engage in individual imagination, joint discussion, and concept sketching to stimulate their creative design and reflection. The goal of this research was to generate user expectations and results that would provide valid insights into new trends in future kitchen design and thus provide direction for design practice.

## 2. Materials and Method

### 2.1. Experiment Design and Materials

The study was conducted in a design studio of a college in Wuxi, China, where a series of seven exploratory co-design workshops were held to collect rich descriptive data. Each workshop lasted around 120 min and consisted of four steps: a short introduction, individual creative acts, group interaction and creations, and discussions. The content and materials of the workshop were primarily designed and directed by the research team (consisting of two senior researchers and two Ph.D. candidates). The workshop materials included some tables, a number of chairs, white sheets of A3 paper, colored stickers, and colored pens—tools to help participants express their ideas. Each workshop used two cameras and an audio recorder to record users’ facial expressions, verbal statements, body movements, participants’ interaction scenarios, and the sketching process.

### 2.2. Participants

A total of 28 participants, 15 men and 13 women, participated in the workshop. All participants were Chinese, aged between 20 and 25 years (mean = 22.96, SD = 1.26). They were college and postgraduate students, representing the younger generation of users in developing countries. All participants have different disciplinary backgrounds (design: 46.5%; humanities and social sciences: 32.1%; science and engineering: 21.4%) and different family types (lives alone: 35.7%; lives with classmates: 32.1%; lives with friends: 17.9%; lives with parents: 14.3%). The participants were divided into seven groups (Groups A—G), each containing four participants, a facilitator, and two recorders (one for video recording and one for photo and voice recording) to record the process. Information on the workshops and participants is provided in Table 1. The researchers’ institution gave ethical approval for the study. Ethical issues considered include anonymity, confidentiality, and informed consent. All participants were asked to sign an informed consent form that described the purpose of the study and explained the risks and relevant emergency procedures. They were informed that the study was confidential and that they had the right to terminate their participation at any time. They were enrolled in this study after signing.

### 2.3. Experiment Procedure

**Figure 1:**
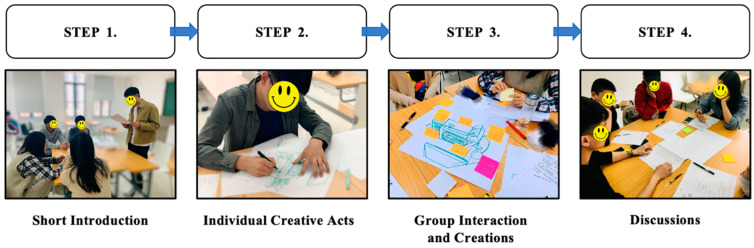
Co-design workshop procedures.

All co-design workshops in this study followed a 4-step process, shown in Figure 1. It is described as follows:

(1) Short Introduction

This phase lasted 15 min. Firstly, the facilitator learned basic personal information about each participant, and the participants had brief conversations with each other, i.e., introduced themselves and discussed their interests, to become acquainted. The facilitator then provided the participants with an introductory document on the workshop’s entire process, describing the workshop’s four steps. Each participant was also asked to imagine future life scenarios (e.g., future clothing trends, future food, future ways of living, and future traveling modes). Finally, participants discussed future life scenarios. They were able to imagine and discuss future life scenarios from future movies and TV shows they had seen.

(2) Individual Creative Acts

This phase lasted 20 min and required each participant to complete it independently. Participants were first asked to recall their personal experiences and stories from their daily lives in the kitchen. They then needed to turn their thoughts to the future and imagine what might happen in the kitchen in the next 5–10 years (in terms of, e.g., preparing ingredients before cooking, cooking dishes during cooking, enjoying delicious food after cooking, and cleaning up after dinner). At the same time, participants were asked to make verbal statements and to record their ideas and thoughts in the form of words or sketches on blank paper. During this process, if a participant was uncomfortable with drawing, they were told that they could express this in text form.

(3) Group Interaction and Creations

This phase lasted 45–60 min. First, each group member shared their ideas and thoughts produced during the “Individual creative acts” step. Afterwards, participants were encouraged to “think outside the box” about kitchen design in terms of, e.g., the problems they wish to solve, their expectations of future kitchen design, health and well-being, intelligent technologies, human–computer interaction, and their experience in the kitchen. Finally, all group members worked together to extend each participant’s creative solutions, during which all participants were free to express their ideas and opinions and jointly sketch their design solutions.

(4) Discussions

This phase lasted 25 min. After the “Group interaction and creations” step, we conducted an unstructured interview and asked participants to review the previous experimental procedures. Each group then selected the most creative and promising future kitchen design solutions. Finally, the researchers asked the participants to explain any user behavior or descriptions that created confusion during the workshop.

### 2.4. Data Collection

The researchers collected all experimental materials created over the entire process, including video and audio material, transcripts, and other materials. Visser et al. (2005) stated that all research processes should be documented, including the manuscript documents drawn by the participants [53]. After each workshop, the researchers kept a content log to record and highlight potentially valuable data for further analysis. Three experts conducted data analysis in the design field in conjunction with co-design workshop records and content logs. Expert 1 had over 26 years of experience in design research and had been involved in user research for over 20 years. Experts 2 and 3 each had 10 years of experience in user research.

### 2.5. Data Analysis

In this study, a qualitative approach was used. The researchers transcribed all video data and audio recordings verbatim during the analysis. Meanwhile, interaction analysis techniques based on identifying verbal and non-verbal phenomena were used to analyze participants’ gestural and postural behavior and their interaction with the materials [54]. The most important part of the data analysis process is coding and classification. The data collected was coded line by line using a coding method that is interpreted as an abstraction of concepts. It assigns general ideas to individual events that occur in the data. The method is a dynamic, intuitive, and creative process of inductive reasoning, reflection, and theorizing. This study is based on open coding and focuses on conceptualizing and classifying phenomena through an intensive analysis of the data collected [55]. Through this process, the researchers reduced the raw information and then identified meaningful patterns and hidden meanings from the data. Finally, the original data were divided into different categories.

## 3. Results

We analyzed the data from the different stages of the co-design workshop. In the phase of individual creative acts, we identified each participant’s creative contributions and mental processes using an interpretive description approach [56] and analyzed the data for the individual. The data were analyzed for the groups’ co-design in “group interaction and creations” and “discussions”.

### 3.1. The Outcome in the Phase of “Individual Creative Acts”

In this phase, we analyzed individual expectations and creativity. In order to improve the objectivity of the measurement, an attempt was made to quantify the qualitative information using coding based on the events described in [57]. Firstly, the participants’ written and verbal statements were segmented and coded by three research experts based on semantic analysis of the Chinese language, while verbal expressions such as “um” and “ah” were omitted [58]. The results were continuously discussed by members of the research team [59], and the verbal statements of each participant in the seven groups were coded using the Am-n format (A denotes group number, m denotes participant number, and n denotes the number of descriptions for each subject). At this stage, a total of 326 original verbal statements were obtained, and the statements without any effective creative design or demand thinking were removed.

A total of 158 qualified verbal statements were eventually used, creating a total of 5 future kitchen design categories, including 13 sub-categories, with the number of qualified verbal statements in each sub-category varying from 5 to 20. These categories include kitchen space layout, kitchen functions and technology, cooking and healthy diets, psychology and emotions, and environmental protection and sustainability. The categories, sub-categories, descriptions of category characteristics, and example descriptions of the participants are shown in Table 2.

In the phase of individual creative acts, the individual’s expectations for the future kitchen leaned towards the needs level, with concerns mainly arising from the difficulties and pain points that the participants themselves experience and encounter when using the kitchen in their daily lives. For example, one participant stated, “The current kitchen takes up too much space. I hope the future kitchen is built-in and hidden in the wall to save space” (B_4-2_).

### 3.2. The Outcome in the Phase of “Group Interaction and Creations” and “Discussions”

During the phase of group interaction and creation, the group members explored the possibilities and innovative solutions for the future kitchen. An example of the process in this phase is shown in Table 3. Participant A1 (in Group A) presented his ideas and thoughts, while the other group members A2, A3, and A4 added to and expanded on A1’s ideas. This process of dialogue demonstrates the co-design process that takes place in the group and that enriches the creativity of A1. During this process, the verbal and nonverbal behaviors performed by the participants were recorded.

**Figure 2:**
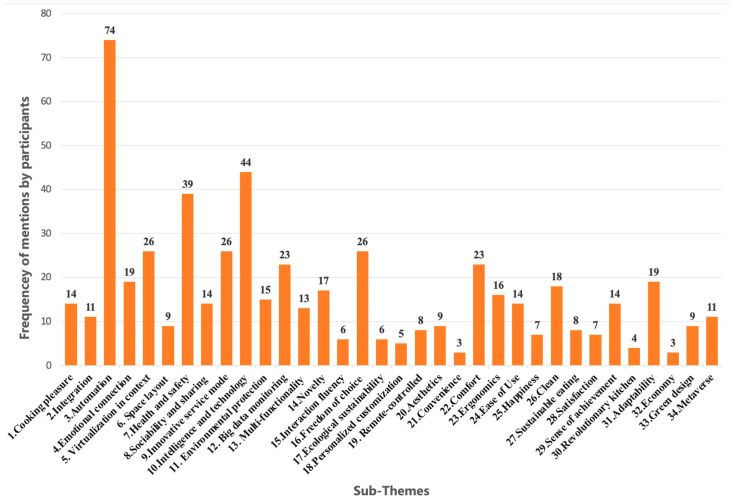
Distribution of sub-themes mentioned by participants (frequency).

Each group produced complex data materials in the phases of “group interaction and creations” and “discussions”. A wealth of collected data needed to be downscaled to search for similarities or differences, find themes, and develop categories. The approach we followed was mainly in line with Pettersson and Karlsson’s user research analysis [60]. At this stage, the focus is on the creativity of each group rather than on individual creativity. Therefore, the participants’ verbal statements were recoded based on Chinese semantic analysis. Each verbal statement was coded with the “An” format (A denotes the group number, and n denotes the number of descriptions for each subject during the joint discussion among all participants). A total of 837 original verbal statements were obtained at this stage, and any descriptions with no valid creative design or demand thinking were removed. Finally, 560 qualified verbal statements were used, ranging from 55 to 113 per group. Each group of materials was collated by three experts and grouped according to the characteristics of the themes, and 34 sub-themes were generated and defined. Table A1 presents the specific characteristics of each sub-theme, qualified verbal statement examples, and participants’ sketches. Figure 2 shows the distribution of sub-themes (frequency).

**Figure 3:**
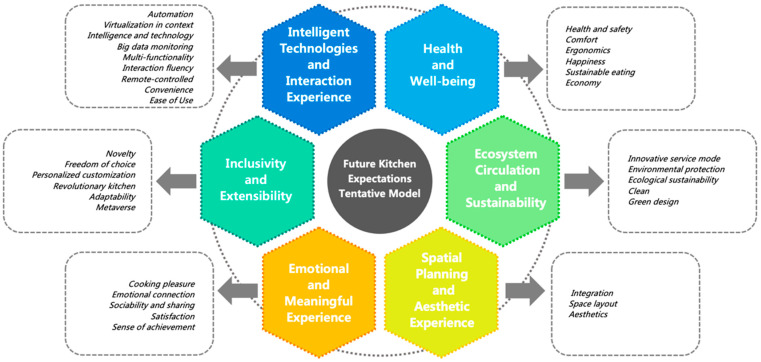
A tentative model of future kitchen expectations for Chinese college students.

In the next stage, the 34 sub-themes were downscaled by the three researchers in this study. The results show that participants’ future expectations are not a single dimension; the clustering into six themes represents the six main directions that Chinese college students expect from future kitchen design. Based on these six themes, a tentative model of future kitchen expectations was constructed as a tool for design researchers and designers to understand user expectations, as shown in Figure 3. The six dimensions of this expectation model include Intelligent Technologies and Interaction Experience, Health and Well-Being, Inclusivity and Extensibility, Ecosystem Circulation and Sustainability, Emotional and Meaningful Experience, and Spatial Planning and Aesthetic Experience.

#### 3.2.1. Intelligent Technologies and Interaction Experience

Intelligent technology has gradually become commonplace in today’s lives, and its emergence has primarily changed the environment, society, economy, and culture in which people live. In the future, intelligent technology will bring about more innovations and changes to our lifestyles, meeting the diverse needs of people and engendering more positive experiences and feelings. The first dimension of the model describes users’ expectations from the technological and usage dimensions of future kitchen design. It is closely related to technological advances such as big data, artificial intelligence, virtual reality, and the emergence of new ways of interaction. This dimension consists of nine sub-themes: automation, virtualization in context, intelligence and technology, big data monitoring, multi-functionality, interaction fluency, remote control, convenience, and ease of use.

Some participants hoped that the equipment in the future kitchen would be fully automated, without commands or manual operation. However, they insist they need to take control of the products and equipment in the kitchen in their role as owners. One respondent stated: “I would like to be able to use machines through voice to help me clean up my bowls and chopsticks, and then help me send them to the washing machine to be washed and placed automatically. The control in this process is up to me, not the machine” (Group A—participant 3). In the context of virtualization, one participant expects a virtual immersion experience: “In the future, we will be able to change the scenes in the kitchen through projections, such as projecting scenes from outer space and wilderness to give us a sense of realistic immersion” (Group A—participant 4).

The participants expect more intelligent kitchen equipment and environments in the future that can monitor their health through big data and provide advice on food pairings. Intelligent technology can also provide more functions to help people with complex everyday cooking tasks. These functions can provide more effective support and provide them with more options to meet their needs. For example, one participant noted: “I can also remotely control the kitchen equipment for cooking when I am not at home. I can control the cooking on my mobile phone, so I can come home and enjoy the food immediately” (Group C—participant 2). In addition, participants wanted more efficient interactions between people and intelligent devices, making it easier to use intelligent products in the kitchen.

#### 3.2.2. Health and Well-Being

The second dimension is the users’ expectations of the kitchen in relation to good health and well-being. Chinese college students expect future kitchen design to focus more on the well-being of individuals, families, and society. This dimension consists of six sub-themes: health and safety, comfort, ergonomics, happiness, sustainable eating, and economy.

Firstly, participants believe that the kitchen should have health and safety attributes and that a healthy diet is the basis and guarantee of human health. COVID-19 causes younger generations to be more aware of healthy diets and food safety. Many participants mentioned that they would like to monitor food quality and that healthy food can help them to remain healthy so that they can enjoy their food with peace of mind. For example, one participant stated: “Providing fresh ingredients with reliable sources and reminders of the shelf life of those ingredients will make my family’s diet more healthy and safe” (Group A—participant 2).

Secondly, the comforts of the kitchen are worth attention. It is essential to pay attention to ergonomic problems in kitchen design, e.g., appropriate sizes and proportions, which will allow for an improved user experience and reduce health problems caused by overwork. The future kitchen will break away from the confines of the area and become a place where people can share joy, increase emotional interaction, and generate a sense of happiness. The participants also look forward to breaking the traditional mindset and developing a new sustainable eating habit by focusing on nutritional matching and healthy eating. One participant noted: “I enjoy exercising, and the future kitchen will offer me a healthy diet based on my current physical composition. It will break the model of eating breakfast, lunch, and dinner daily and change it to new sustainable eating habits, such as eating smaller portions and more frequent meals” (Group E—participant 1).

Thirdly, there is also a need to consider economic issues and whether the future kitchen can offer an affordable way to avoid unnecessary expenditures for individuals and families and waste resources for the economy, society, and the environment. Users expect the future kitchen to be an optimal solution that meets their needs and is economical and practical (Group F—participant 3).

#### 3.2.3. Inclusivity and Extensibility

This dimension consists of six sub-themes: novelty, freedom of choice, personalized customization, revolutionary kitchen, adaptability, and the metaverse. The future kitchen should not be limited by time and space and should be conceptualized as a space that is without boundary restrictions and that is open and transregional. The data show that participants are looking for a novel and revolutionary cooking experiences in future kitchens. Regarding novelty, one participant stated: “When there is a lack of food ingredients, the desired taste can be achieved by combining and matching existing food ingredients” (Group D-participant 4). Another participant also expressed a new form of revolutionary kitchen: “Could the future kitchen be transferred to the outdoors? There is no fixed location, and a new form of an outdoor kitchen can be formed anywhere” (Group C—participant 1). Many participants wanted to have more control in future designs, to change them at any time according to their needs, and to take full advantage of their autonomy. One respondent stated: “I leave the tiring process of washing and cleaning up to the machine, while I enjoy the fun and process of cooking” (Group A—participant 1). The future kitchen will also need to focus on the specific needs of each individual and provide personalized customizations. For example, intelligent technology for kitchen design can identify and analyze user data and provide personalized solutions: “In the future, I would like to install an intelligent sensor on my kitchen wall at home that can automatically detect my health and provide nutritional advice” (Group B—participant 1).

The future kitchen design will also need to be adaptable to allow for proper use by a wider group of users and reduce the number of problems. One participant noted: “I want a kitchen in my house in the future that can be used by young people, children, and older people. Anyone can use it without difficulty” (Group D—participant 2). Furthermore, participants want the future kitchen to be more diverse, breaking away from the current format to form new types of virtual and real space. Some participants stated their intention to use the current hot concept of the ‘metaverse’ in the design of future kitchens.

#### 3.2.4. Ecosystem Circulation and Sustainability

This dimension is concerned with the long-term development of the world of human existence. It includes five sub-themes: an innovative service mode, environmental protection, ecological sustainability, and clean and green design. As human society faces environmental, cultural, economic, and sustainability issues, future kitchen design needs to focus more on environmental protection, green design, and sustainability.

With the development of science and technology, such as big data and the Internet of Things (IoT), lifestyles have changed to a great extent. Participants hope that a new sustainable and innovative service mode can emerge in the future kitchen based on big data and IoT technology: “Users place their orders online, and merchants, suppliers, and other stakeholders provide a more efficient service by providing users with ingredients delivered to their doorstep”. This innovative mode also considers combining mass production, industrialization, and on-demand distribution of fresh food materials to provide a sustainable service.

The participants also attach great importance to the issue of clean environmental protection. Kitchens generate much garbage, which, if not disposed of properly, can place a significant burden on the environment and create pollution that is not conducive to ecological recycling and sustainability. Therefore, future kitchen designs should focus on green design and provide more ecologically sustainable design solutions. For example, one participant stated: “In the future kitchen, there will be an energy regeneration machine that processes kitchen waste into renewable energy and transports it to where it is needed, such as pig farms” (Group D—participant 3). This description reflects users’ expectations for sustainable resource recovery and energy regeneration in the design of future kitchens.

#### 3.2.5. Emotional and Meaningful Experience

The fifth dimension is “emotional and meaningful experience”, which relates to users’ emotions and the meaning of life. It includes five sub-themes: the pleasure of cooking, emotional connections, sociability and sharing, satisfaction, and a sense of achievement. In this dimension, participants indicated that they would enjoy the cooking process and the pleasure it brings them. One participant stated: “In the future kitchen, making cooking a hobby [of mine] and cooking food that my family will enjoy will reflect my values” (Group E—participant 1).

The participants expect the future kitchen to be a space that enhances emotional connections between people and promotes family well-being. Some participants associate the taste of food with emotional memories, reflecting deep emotional bonds in the family. Furthermore, the future kitchen will become a new social platform to share life with friends, strengthen emotional connections, and lead to more meaningful lifestyles.

One participant mentioned: “I often have all sorts of complex jobs in the kitchen, which frustrates me and makes me lose confidence. Will the future design build my confidence and make me feel good about my cooking skills?” (Group G—participant 3). This description demonstrates the participants’ desire for confidence and a sense of achievement in the kitchen. The sub-theme “satisfaction” reflects the participants’ desire to reflect their values and sense of meaning in kitchen activities, to gain recognition and approval from others, and to achieve psychological satisfaction.

#### 3.2.6. Spatial Planning and Aesthetic Experience

The final dimension is “spatial planning and aesthetic experience”, which consists of three sub-themes: integration, space layout, and aesthetics. Many participants expect a more rational design of the future kitchen’s overall space, with more integrated kitchen appliances and equipment. The future kitchen can become a holistic system, through which every item therein can be controlled in a more orderly and planned way. One participant noted: “ I hope the future kitchen will be a large integrated system through which I can freely control any device in the kitchen”.

At the same time, the participants wanted a neat, clean, and organized kitchen space. Each area of the kitchen needs to be carefully planned and designed. For example, one participant noted: “I hope the future kitchen environment will not be messy, the layout will be more reasonable, and kitchen appliances are systematized and integrated” (Group B—participant 4).

In addition, participants also expect a more attractive decorating style and color scheme to meet their aesthetic needs. For example, one participant noted: “I hope that I can define the decoration style and color scheme in the future kitchen. The kitchen could present different environments in different seasons” (Group F—participant 2). Participants said that advanced technology is needed to provide users with a more personalized aesthetic experience with customized solutions. It would enhance the aesthetic experience of the users and give them a sense of aesthetic pleasure. Users hoped for aesthetically pleasing environments that matched their moods automatically.

## 4. Discussion

This study used the co-design method and brought design researchers and college students together in a workshop to explore Chinese college students’ expectations of trends in future kitchen design, with an emphasis on health and well-being. We found several innovations and differences by comparing our findings with previous research.

We wanted to explore the relationship between co-design and kitchen design research. Using the co-design method, participants were given a space to express and reflect through four steps of engagement, and they developed their ideas of a future kitchen design in both the individual and co-design stages. We gained a wealth of insights and data that led to the generation of 34 creative sub-themes and the construction of a six-dimensional user expectation model. This study expands and innovates on co-design in terms of methodology. By remaining open to the process of co-design, we focus not only on solving current user demand but also on prospective user studies to stimulate and understand users’ expectations and insights for the future. During different steps of the workshop, we used different methods of data acquisition and analysis, with different roles between the layers of analysis. In the “individual creative acts” step of the workshop, it was understood that building trust among participants and creating a safe space for open discussion is the “foundation” of co-design. The participants were then asked to think freely about the future kitchen design without interrupting each other, allowing for creativity and imagination. This differs from previous studies of co-design, which have tended to involve direct multi-stakeholder co-discussion and design, resulting in the restriction of individual ideas and insufficient expression [48,50,61]. The expression of individual innovation is the basis of co-design and provides individual materials for a creative co-design pool, which is indispensable. In the “group interaction and creations” step, we asked participants to sketch their ideas. Previous studies have shown that using such materials in co-design workshops facilitates participants’ creative insights and allows them to engage more actively and interestingly with the tasks [52,62,63]. During this step, the ideas presented by the more active participants (group leaders) were able to drive and activate the creativity of other group members, stimulating ideas that they would normally struggle to activate themselves (stimulating weak connections that would normally be difficult to retrieve). In some cases, the group leader would also guide the direction of ideas. The difference from previous studies is that, in this study, college students thought of more innovative and creative solutions. The co-design method in this study thus promoted group creativity and provided greater insight into the feelings and perceptions of Chinese college students regarding the health and well-being of future kitchen design.

The relationship between the tentative new model and health promotion also deserves further exploration. The new model constructed in this study focuses on users’ expectations of kitchen design in the context of the COVID-19 pandemic. Health and well-being in the kitchen have always been key concerns, and “health” in the kitchen is a broad concept. This research aimed to study the health and well-being of the kitchen in the special context of the COVID-19 pandemic. The concept of “health” here includes not only daily health but also the role played by the kitchen on certain occasions. For example, cooking to address health problems during times of illness will be different from cooking on a daily basis. Therefore, the new model provides us with more topics to focus on in terms of health promotion, such as “Health and Well-Being” which focuses not only on the basic elements of daily kitchen use, i.e., health and safety, comfort, and ergonomics, but also on the future. It also focuses on “Sustainable Eating”, which will play a more positive role in promoting our health. In terms of “Inclusivity and Extensibility”, users hope not only that the future kitchen will be personalized but also that it will be adaptable to a wider range of people and will be able to respond to more specific situations. The new model provides additional health-promoting elements that can be used as a reference for future kitchen design. At the same time, the health of users can be promoted, thereby verifying the value and practical implications of the new model.

When we considered the participants’ background information and the characteristics of the different groups, new and interesting findings emerged.

The difference in the gender composition of the participants in the groups affects the expectations of the future kitchen. Participants in different groups had different gender collections. Groups A, B, and G were mixed with male and female members. Groups C and E were all male, and Groups D and F were all female. The all-male groups focused more on the dimensions of intelligent technologies and interaction experiences, inclusivity, and extensibility. They paid more attention to the progress of intelligent technologies and novelty in future kitchen design than the other groups, especially in terms of automation, virtualization in context, intelligence and technology, and freedom of choice. For example, C2 noted, “In the future, when I come home, the equipment in my kitchen can automatically recognize that I am back and start cooking food based on my physical condition today”. The all-female groups focused more on health and well-being as well as emotional and meaningful experiences, specifically comfort, ergonomics, sustainable eating, and emotional connections. D3 hopes to enjoy the company of families in the kitchen and have a more comfortable experience during the cooking process. The mixed groups had more divergent thinking and more diverse expectations of the future kitchen, paying more attention to and looking forward to multiple dimensions. This illustrates that the involvement of users from multiple genders and backgrounds leads to more creativity and more diverse values during the co-design process.

We also found many similarities between the research results and the characteristics of Chinese college students. The dimension with the highest frequency was intelligent technologies and interaction experience. This is consistent with previous research, where young people have always been recognized as the driving force behind new cultural trends [9]. College students, a subset of young people, have a high level of acceptance of new ideas and technologies. Some participants hoped that the kitchen equipment and environment could monitor their physical health using big data and provide suggestions on food combinations. They were also more concerned about health issues and had a greater desire for a higher level of health literacy. In addition, the dimension of emotional and meaningful experience also demonstrated their emphasis on social relationships and the emotional issues of family.

We found that the six dimensions of user expectations for future kitchen design were difficult to consider separately.

Previous studies have shown that human needs are difficult to satisfy from a single dimension, and “need” is a highly abstract concept that is difficult to accurately target [64]. In general, from a system perspective, the six dimensions are closely related to each other and cannot be considered in isolation. Firstly, the dimension of “intelligent technologies and interaction experience” expresses the user expectation that advanced technological developments meet the technological requirements of the future kitchen. Intelligent technologies can meet expectations in terms of health and well-being through technical support. As one participant mentioned, “I would like to see smarter kitchen equipment and environments in the future that can monitor my health through big data and give suggestions on my diet”. Secondly, the development of intelligent technologies can also respond to more expectations in the kitchen in the dimensions of inclusivity and extensibility, as well as emotional and meaningful experience. Thirdly, emotional and meaningful experiences are inextricably linked to health and well-being. Ecosystem circulation and sustainability are an essential concern for the development of human society in the face of complex social and environmental sustainability challenges and profoundly affect the health and well-being of humankind. In addition, “emotional and meaningful experience” is closely related to the spatial planning and aesthetic experience of the future kitchen.

This research has some limitations that need to be addressed in future research. Firstly, according to the method of co-design adopted in this paper, more stakeholders in kitchen design, such as business executives, government officials, interior designers, and intelligent appliance designers, should be invited to participate in the next round of research on co-creation for more insight. Next, our proposed tentative model of user expectations may have limitations in practical application, and we need additional investigation and empirical research to verify and evaluate its validity and value. Furthermore, this paper briefly analyzes the differences in expectations between different groups of participants, which still needs further research. We will further engage participants in a collaborative experiment in a real kitchen environment to measure users’ expectations.

## 5. Conclusions

This study focused on kitchen design. User research was conducted, and a tentative model of future kitchen expectations was derived through qualitative research methods. In the context of the COVID-19 pandemic, the new model provides diversified strategic suggestions for future kitchen design from a multidimensional perspective, including intelligent technologies and interaction experiences, health and well-being, inclusivity and extensibility, ecosystem circulation and sustainability, emotional and meaningful experiences, and spatial planning and aesthetic experiences. Specifically, in the first dimension, there is an expectation that future kitchen design will lead to intelligent, more efficient, and more convenient lifestyles. Next, in terms of health and well-being, people expect the future kitchen to deliver value and provide tangible improvements in mental and physical health and well-being. The third dimension, inclusivity and extensibility, embodies more possibilities and novelties in future kitchen design, offering inclusive design solutions for a wider range of people. The fourth dimension considers the environmental and ecological sustainability of human existence. In the fifth dimension, the future kitchen needs to be an experiential space that carries emotional significance and meets users’ expectations in terms of psychological feelings. Finally, the dimension of spatial planning and aesthetic experience is a reflection of the environmental, aesthetic, and perceptual aspects of the future kitchen that will transform users’ living spaces. The new model presents specific design strategies and elements in these six areas that can address existing complexities and guide future innovations in kitchen design with respect to health and well-being. As a result, it can improve health and well-being in a more systematic and effective way.

The results provide valid insights into the early stages of design, as users’ speculations and expectations of future design can be built into these early stages, providing valuable information for the design of the future kitchen in terms of health and well-being. The tentative model of future kitchen expectations we have developed can be used as a guide for the development of a systematic design strategy for future kitchens. The future kitchen, in order to be adopted by younger generations, needs to positively affect users’ lives and improve health and well-being. The approaches adopted in this study can also be used to explore other areas of design research and obtain vital user data to guide forward-looking user research.

## Figures and Tables

**Table 1:** Workshop and participant information.

Workshop	Group A	Group B	Group C	Group D	Group E	Group F	Group G
Participants	4	4	4	4	4	4	4
Discipline	4 Design	4 Design	1 Design 1 Art 1 Psychology 1 Engineering	1 Psychology 2 Art 1 Design	1 Engineering 3 Computer science	2 Design 2 Management	1 Engineering 1 Design 1 Psychology 1 Management
Sex	3 Male 1 Female	2 Male 2 Female	4 Male	4 Female	4 Male	4 Female	2 Male 2 Female
Mean age	23.75 years	23.25 years	23.5 years	24.25 years	21.5 years	22.75 years	23.25 years
Household type	2 Lives with classmates 2 Lives with parents	2 Lives with friends 1 Lives with classmates 1 Lives with parents	2 Lives alone 2 Lives with classmates	2 Lives alone 1 Lives with friends 1 Lives with parents	2 Lives with classmates 2 Lives alone	2 Lives alone 1 Lives with classmates 1 Lives with friends	2 Lives alone 1 Lives with classmates 1 Lives with friends
Cooking frequency	A1 sometimes A2 occasionally A3 sometimes A4 often	B1 often B2 often B3 sometimes B4 sometimes	C1 hardly ever C2 often C3 sometimes C4 sometimes	D1 always D2 often D3 hardly ever D4 sometimes	E1 never E2 sometimes E3 hardly ever E4 sometimes	F1 sometimes F2 often F3 often F4 occasionally	G1 often G2 sometimes G3 hardly ever G4 sometimes
Workshop Duration	120 min	136 min	125 min	122 min	105 min	127 min	113 min

**Table 2:** Category descriptions.

Categories	Sub-Categories	Category Characteristics	Description Examples
Kitchen space layout	Design layout	Consider the space, environment, and layout design in the kitchen	B_4-2_: I think the current kitchen takes up too much space, and I hope that in the future the facilities in the kitchen will be a form of built-in, hidden in the walls to save space.
	Integration	Kitchen appliances, equipment, and facilities will be integrated, and the cooking process will be more rationalized	A_1-2_: I want the appliances inside the future kitchen to be integrated.
	Comfort	The overall environment and facilities of the future kitchen will give the user a more comfortable experience	F_2-3_: Could the future kitchen make me feel more comfortable and relaxed as soon as I walk into it?
Kitchen functions and technology	Automation	The future kitchen will have increased automation and more assistive technologies	D_4-1_: In the future, the machine will automatically process the ingredients before cooking, and deliver them to me when they are ready.
	Multi-functionality	The future kitchen will have more new and varied functions	B_3-7_: I hope that the sink will be more versatile in the future kitchen, such as filtering and self-cleaning functions.
	Big data monitoring	Information monitoring, analysis, and optimal solutions through big data	B_2-2_: The body’s health information is monitored through big data technology, and individual ingredients are matched according to health status.
	Smart technologies	Smarter cooking equipment, appliances, and environments	D_1-1_: I would like to have a smart assistant inside my kitchen that can help me with my cooking.
Cooking and healthy eating	Health	Pay attention to a healthy lifestyle for the future	A_2-2_: I expect the future kitchen to be free from grease and fumes and the hassle of scrubbing and cleaning. At the same time, the ingredients of the future are reliably sourced, healthy and safe.
	Safety	Focus on safety and health issues in the kitchen diet	E_3-3_: When I cook in the future, I hope that the quality and safety of the food will be guaranteed, which will give me great peace of mind.
Psychology and emotions	Enjoyment	Enjoy the process of cooking and the fun it brings	A_1-1_: I think cooking will be a fun and enjoyable experience in the future.
	Emotional connection	Expectations related to human emotions in the future kitchen	C_2-7_: I hope to be able to eat with distant families and reduce the loneliness of living alone.
	Sociability and Sharing	Kitchen events have become a new way to share life and socialized	A_2-6_: In the future kitchen, a gathering of friends and relatives can be simulated by technology.
Environmental protection and sustainability	Environmental protection	Focus on healthy and hygienic kitchens and a tidy environment	C_4-6_: After cooking, we need to pay attention to the tidiness of the kitchen environment, cleaning up and separating the waste.

**Table 3:** An example of the Group Interaction and Creations process.

Participant	Verbal Behaviors	Nonverbal Behaviors
A1	In the future, I want kitchens to be virtual scenarios that can simulate cooking with distant family members.	Looking at the facilitator to express his views while gesturing in the air with a pen.
A2	I think your idea is very interesting, I often miss my parents as we don’t live in the same city. I would very much like to achieve an emotional connection between families in the future by being in a virtual space.	Looking at A1 while describing
A3	I still want to be able to cook and eat with my family in real scenes.	Looking at A1 while describing
A4	I know that the concept of ‘metaverse’, which has recently emerged, seems to allow for this emotional connection, combining real and virtual scenarios. I believe that in the future, technological advances will make this possible. In the metaverse, each person can have their own identity, and through this virtual socialization, an emotional connection can be made.	The participant was very excited and eager to convey the concept of the “metaverse” to everyone.
A1	Yeah, I think the concept of “metaverse” is quite good, but I don’t understand it.	Show a supportive attitude.
A3	However, the “metaverse” concept is still relatively new, and it is unclear what kind of technology will be used to realize it in the future.	Looks very confused.
A4	I am sure it will be possible in the future so that the boundaries of time and space can be broken and an emotional connection between people can be established.	Looking at A3 and expressing his opinion with confidence and certainty.
A1	I am particularly bullish on metaverse and virtual reality technology. I hope to have the feeling and experience of having my friends and family around me. It makes me feel warm.	Looking at A3 and A4 for expression.
A1	In the future, I want to be able to cook and communicate with my parents or friends in the kitchen through virtual screen projections (Sketch while describing it).	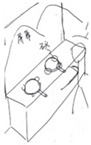
Facilitator	I thought everyone’s ideas were very interesting, and I hope you can think outside the box.	Express this while simultaneously recording critical information on paper.

## Data Availability

Not applicable.

## Appendix A

**Table A1:** Sub-themes descriptions.

Sub-Themes	Sub-Themes Characteristics	Qualified Verbal Statements Examples	Participants’ Sketches
1. Cooking pleasure	Enjoy the process of cooking and the pleasure it brings	A3 Cooking will be a fun and enjoyable experience in the future, and I am happy to do it.	
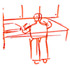
2. Integration	The future kitchen environments, equipment, and cooking processes will be integrated	B7 The future kitchen will be a holistic system where all the equipment is integrated, and I can follow the process to do cooking without worrying about it being messy.	
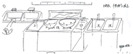
3. Automation	The level of automation in future kitchens will increase, and more automated assistive technologies will emerge	D20 I want machines in future kitchens to match the amounts to the ingredients needed for cooking and automatically create the meal I want.	
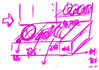
4. Emotional connection	Expectations related to human emotions and moods in the future kitchen	D7 In the future, I want a machine that can connect families and friends as an emotional bond, bringing families closer to each other.	
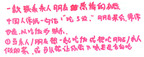
5. Virtualization in context	Expanding the user’s sense of reality and integrating virtual scenarios into real scenarios	B63 I want the ambience of the future kitchen to be adjustable, with projected virtual environments and scenes.	
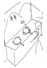
6. Space layout	Consider the design of the space, environment, and layout of the future kitchen	B50 In the future, the kitchen may be open-plan, and there will be no boundaries between the kitchen and the living room, which will be connected.	
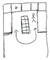
7. Health and safety	Pay attention to the future of healthy lifestyles and food safety	A16 Ingredients are reliably sourced, healthy and safe, and in the future, there will be space in the kitchen to store food and record relevant data.	
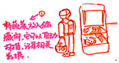
8. Sociability and sharing	Kitchen events have become a new way to share life experiences and socialized	G23 I hope that a community kitchen can be set up in the future to create connections between people in the community. People can get together and socialize, share ingredients, meals, etc.	
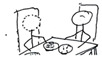
9. Innovative service mode	Future Kitchen offers more innovative and sustainable services and distribution models	A39 In the future, there will be a new form of meal delivery where the user places an order at home, and the background system automatically confirms it and delivers it to users.	
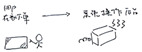
10. Intelligence and technology	Smarter cooking equipment, cooking appliances, and cooking environments	B22 In the future, intelligent devices will alert people that the meal is ready and automatically serve the food.	
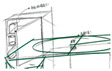
11. Environmental protection	Concern about the messy, healthy, and hygienic problems in the kitchen and issues of pollution	A8 Intelligent equipment in the kitchen that allows for waste degradation and does not have an environmental impact.	
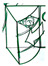
12. Big data monitoring	Information, data analysis, and optimal solutions through big data	B54 Big Data records each person’s data, stores user data centrally, collects and analyzes it, and matches ingredients to each person’s health status.	
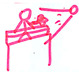
13. Multi-functionality	The future kitchen is given new and varied functions	F60 I wish there were a function to “design the taste of the dish”. For example, how much salt to add? How much oil to add? The order in which the dishes are added, the heat level, etc.	
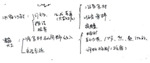
14. Novelty	Looking for an unprecedented cooking experience and novelty	A19 The future kitchen is a large virtual glass space with a human cooking environment inside.	
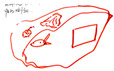
15. Interaction fluency	The future kitchen will have more fluent, natural, and intuitive interaction, and complexity will be reduced	E30 The operating interface of future intelligent products can automatically recognize and select functions through a more straightforward operation.	
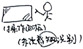
16. Freedom of choice	In the future kitchen, users will have a high degree of freedom of choice when it comes to cooking and interacting with the equipment.	B75 During the cooking phase, the pan can be selected in different modes according to the user’s needs, whether it cooks automatically or the user does it himself.	
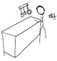
17. Ecological sustainability	The future needs to focus on the health of the internal kitchen environment and the ecological sustainability of the external human society associated with the kitchen.	D12 There will be food waste collection and recycling machines in the kitchen, which are processed and automatically recycled to where they are needed, such as pig farms. It is a renewable energy treatment and sustainable recycling of resources.	
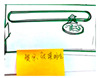
18. Personalized customization	Personalized solutions based on the specific individual needs	B18 In the future, ingredients and kitchen environments will be personalized according to users’ habits, health, and nutritional needs.	
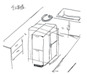
19. Remote-controlled	In the future, it will be possible to issue commands to appliances in the kitchen remotely and control them whenever and wherever the user wants	B80 Before you leave home, put the ingredients in through the smart machine entrance, and the machine provides an automatic keep-fresh function so you can go out to work. Before you return from work, select the cooking function on the mobile app, and the smart machine works automatically so you can enjoy your meal immediately when you get home.	
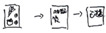
20. Aesthetics	Consider the color scheme and aesthetics of the kitchen environment, food, and food presentation	E34 I hope the future kitchen is minimalist and aesthetically pleasing, with a brighter, more vibrant color scheme. At the same time, the food presentation looks pleasing on the plate.	
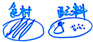
21. Convenience	Cooking activities in the future kitchen will become more accessible and more efficient, with more convenient equipment functions	F14 The future design starts with convenience as a critical point, wanting to make activities in the kitchen convenient and easy.	
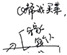
22. Comfort	Focus on the comfort of the future kitchen to reduce unpleasant feelings	D42 In the future, I will be very relaxed when moving around in the kitchen without worrying about doing something wrong and causing trouble and distress.	
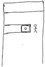
23. Ergonomics	Pay attention to space size, rational operation, and ergonomic issues in kitchen design	F15 I hope that the height and size of cabinets in the kitchen work area will be designed to consider the different age groups of users and whether they are in proportion to their size.	
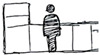
24. Ease of Use	The equipment and appliances in the kitchen are simple and easy to use	A29 In the future, when operating the fridge interface in the kitchen, I will not be misled into being unable to find the options and content I want.	
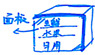
25. Happiness	The future kitchen can bring a sense of well-being to users	F55 When I enjoy a meal in the kitchen, it is as if it has been prepared by families, bringing me a feeling of home and happiness.	
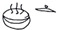
26. Clean	The future kitchen cleaning, hygiene, and waste removal	G36 The future kitchen will be spotless, with fewer fumes, and easy to clean, and the food waste will be cleaned up automatically.	
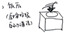
27. Sustainable eating	To be able to enable healthier and more sustainable eating	F43 In the future, I want a healthier diet that I can maintain over time. Change my eating pattern and enjoy fresher, more varied food for less money.	
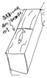
28. Satisfaction	The future kitchen design can give users a sense of self-satisfaction	F55 In the future, the kitchen will be able to fulfill my needs immediately when I want something so that I will be very happy hahaha...	
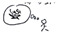
29. Sense of achievement	Bring a sense of self-achievement and build confidence when using the kitchen	G11 I often have a variety of complicated tasks in the kitchen that often leave me frustrated and lose my confidence. Can future design give tips on building my confidence and feeling good about my cooking skills?	
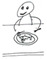
30. Revolutionary kitchen	The radically transformative kitchen design of the future	C79 Can the future kitchen not be set in the home? My future kitchen could be in a separate area outside the home, one such area on each building floor.	
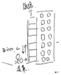
31. Adaptability	The future kitchen will be suitable for different age groups of users	F16 There could be two working areas inside the kitchen, one for adults and the other for children. Ideally, it would also be possible for older people to use it. Will this be possible in the future kitchen?	
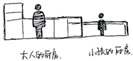
32. Economy	Affordable and adaptable kitchen design that makes the most of resources	D74 Can the future kitchen reduce the waste of social resources? Avoid unnecessary features and space. Maximize the use of resources and design a new economical and practical future kitchen.	
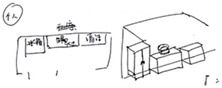
33. Green design	Future design solutions in the kitchen that are more sensitive to human health and green	D7 In the future, kitchens will have a small, hidden, integrated vegetable garden inside the cabinets. It can be grown without pollution and produce fresh, nutritious, healthy, hygienic vegetables. I can eat them as they are picked, which gives me great peace of mind.	
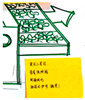
34. Metaverse	The future lifestyle will be a new form of deep integration of virtual and real, looking forward to the emergence of the “metaverse” concept space in the kitchen	C27 In the future, I want to feel a fully immersive experience in the kitchen, a ‘metaverse’ environment where I can share freely and socialize with people of different identities.	
